# Bridging Strategies to Allogeneic Transplant for Older AML Patients

**DOI:** 10.3390/cancers10070232

**Published:** 2018-07-11

**Authors:** Judith Hecker, Isabella Miller, Katharina S. Götze, Mareike Verbeek

**Affiliations:** Department of Medicine III, Hematology and Oncology, Technische Universität München, 80333 Munich, Germany; judith.hecker@tum.de (J.H.); isabella.miller@tum.de (I.M.); katharina.goetze@tum.de (K.S.G.)

**Keywords:** elderly AML, bridging strategies, allogeneic stem cell transplant, chemotherapy, hypomethylating agents

## Abstract

Treatment options for older patients with intermediate or high-risk acute myeloid leukemia (AML) remain unsatisfactory. Allogeneic stem cell transplantation, the treatment of choice for the majority of younger AML patients, has been hampered in elderly patients by higher treatment related mortality, comorbidities and lack of a suitable donor. With the higher availability of suitable donors as well as of reduced intensity conditioning regimens, novel low intensity treatments prior to transplantation and optimized supportive care, the number of older AML patients being successfully transplanted is steadily increasing. Against this background, we review current treatment strategies for older AML patients planned for allogeneic stem cell transplantation based on clinical trial data, discussing differences between approaches with advantages and pitfalls of each. We summarize pre-treatment considerations that need to be taken into account in this highly heterogeneous older population. Finally, we offer an outlook on areas of ongoing clinical research, including novel immunotherapeutic approaches that may improve access to curative therapies for a larger number of older AML patients.

## 1. Introduction

Acute myeloid leukemia (AML) occurs in children and adults at any age, but it is primarily a disease of the elderly, most frequently diagnosed at a median age of 72 years [1]. During the entire lifetime acquisition of somatic mutations leads to clonal hematopoiesis and pre-leukemic lesions, which continuously increase the risk for acute leukemia with age [2,3,4]. Due to the dramatically growing population of people beyond 60 years worldwide whose number is expected to double again until 2050 [5], AML is even now a common disease with an incidence of 100/100,000 inhabitants regarding this elderly population [1].

Meanwhile, age is one of the strongest risk factors for poor outcome in AML. Beside the difficulties in treatment of elderly AML patients due to comorbidities and reduced performance status, poor outcome is caused by a different disease biology with more frequent aberrant cytogenetics and often emerging secondary to myelodysplastic syndromes (MDS) or myeloproliferative neoplasms (MPN) [6,7]. As shown by Appelbaum et al., the number of AML patients with unfavorable cytogenetics and multidrug resistance increases significantly in patients older than 75 years [8]. Therefore, the only treatment with curative intent for the majority of elderly AML patients is allogeneic hematopoietic stem cell transplantation (allo-HSCT).

Although major improvements have been achieved in the outcome of older AML patients undergoing allogeneic HSCT, notably by reduced intensity conditioning regimens and optimization of supportive care, there is a significant gap remaining between patients in need of and those actually finally receiving transplant [9,10,11,12]. Reasons for this are diverse, but include over-estimation of risk for allogeneic transplant in the elderly as well as losing patients on the road to allogeneic transplant due to severe infectious complications as well as relapsed or refractory disease. In an effort to overcome these limitations, bridging strategies beyond conventional chemotherapy and reduced-intensity conditioning regimens have been employed. In this article, we aim to give a comprehensive overview on strategies of how to identify eligible patients in this highly heterogeneous older AML population and how to successfully bridge them to allogeneic stem cell transplantation.

## 2. Pretreatment Considerations (Patient Identification)

Consistent with the indication for younger AML patients, the decision to perform allogeneic HSCT within the elderly AML patient cohort is based on assessing the risk to benefit ratio for long-term survival which takes into account the reduction of the risk of relapse vs. the non-relapse mortality (NRM) [13,14]. Besides cytogenetics and molecular genetics as stratification tools for the identification of intermediate or high-risk AML as indications for allogeneic HSCT, the ratio also includes patient-related factors—such as performance status, graft and donor source or disease status (e.g., the remission state) [15].

In general, allogeneic HSCT has shown a survival advantage when the risk of relapse exceeds 35–40% which affects the majority of elderly AML patients considering the higher incidence of poor prognostic factors. Elderly AML more often arises as secondary leukemia (sAML) from antecedent myelodysplastic syndromes (MDS), myeloproliferative neoplasms (MPN), or after chemotherapy or radiation as therapy-related AML. In particular, older AML patients are more likely to present with poor-risk cytogenetic features such a complex or monosomal karyotype [16], which show a low rate of remission in response to conventional chemotherapy. In comparison to younger AML patients, data on molecular genetics of intensively treated elderly AML patients is scarce [17,18,19]. A recent report on 151 AML patients aged ≥75 years (the majority of which were characterized as de-novo AML) showed a median number of four mutated genes per patient with a spectrum distinct from that of younger AML patients [20]. Gene mutations found with a higher frequency than in previously studied younger patient cohorts were *TET2*, *SRSF2*, and *ASXL1*. Eighty-three percent of AML patients in this cohort harbored at least one gene mutation known to be involved in age-related clonal hematopoiesis [2,4]. However, impact on survival was predicted mainly by poor-risk cytogenetics. The presence of IDH1 mutations (seen in 9% of patients) seemed to confer inferior survival while no clear conclusions could be drawn for other mutations. Thus, the impact of genetic mutations on the outcome of intensively treated elderly AML patients requires further investigation.

Especially for the high-risk AML subgroup, it is recommended to proceed to transplant as soon as first complete remission (CR1) has been achieved [21]. Data analyzed by Röllig et al. derived from the AML96 trial clearly outlines a fatal outcome for high-risk, but fit elderly AML patients not undergoing allo-HSCT with a three-year overall survival (OS) rate of 3.3% [22]. Results from the AMLSG trial AML HD98-B analyzed by Fröhling et al. unambiguously determined high-risk cytogenetics as well as age above 70 years as major risk factors for a poor outcome of elderly AML patients [23]. In contrast, several other studies demonstrated that age by itself should not be a contraindication to transplantation and has an acceptable risk of NRM and no significant impact on OS [24,25,26]. Furthermore, allo-HSCT still remains the only curative treatment option for primary refractory or early relapsed disease, also in the older AML patient cohort [27]. Incorporating risk scores such as the HCT-specific comorbidity index (HCT-CI) score, the disease risk index (DRI), the European Group for Blood and Marrow Transplantation (EBMT) score, and the pre-transplantation assessment of mortality (PAM) risk score can help to individually determine transplant candidacy for AML patients since a good performance status is tremendously important for a good outcome after allogeneic HSCT [15,28,29,30].

The risk of relapse predicted by genetic risk stratification needs to be seen in relation to the risk of transplant-related death. Obviously, the non-relapse mortality (NRM) is higher in an older population, but with the implementation of reduced intensity conditioning (RIC) allowing transplant at advanced age it turned out that chronological age is a far weaker predictor than the patient’s condition. Data analyzed by McClune et al. of over 1000 AML and MDS patients undergoing RIC or non-myeloablative allo-HSCT showed no significant influence of age on NRM, though there was a trend for a higher NRM in AML patients with rising age since NRM at one year was 21% for patients aged 40–54 years, 24% of patients aged 60–64 years, and 30% for patients aged ≥65 years, respectively. However, foremost, a Karnofsky performance score < 80 clearly had negative impact on NRM and OS [25].

In a prospective study by Sorror et al. with 372 patients of 60–75 years, age had no impact on outcome. In contrast, a HCT-specific comorbidity index (HCT-CI) score of 0 compared to an intermediate score of 1–2 or a high risk score of 3 or greater was associated with better survival [24].

The HCT-CI score was developed in 2005 by Sorror et al. to assess transplant related mortality risk for all age groups. It is a predictive score incorporating cardiac, pulmonary, renal, hepatic, gastrointestinal, oncologic, psychiatric, rheumatic, metabolic, vascular, and infectious comorbidities. Each comorbid condition was weighted from 1 to 3 points according to its risk of NRM assessed in a training cohort. A HCT-CI > 2 indicated a two-year-NRM of above 40%, a score of 0 revealed 14% [28]. The predictive power of the HCT-CI score was confirmed in several additional studies [31,32,33]. Taking the increasing number of elderly patients undergoing allo-HSCT into account, it was attempted to incorporate age into a combined HCT-CI/age score. The retrospective data the combined score is based on revealed that age is a risk factor for increased NRM, but beyond the age of 40 years no significant differences between groups of 40–49 years (*p* = 0.17), 50–59 years (*p* = 0.71), and ≥60 years of age were determined. Therefore, age >40 years was counted with 1 additional point. The c-statistic estimates for NRM were better than using the HCT-CI score alone, but not in a statistically significant way (*p* = 0.56), whereas the combined score was a far stronger predictor than age alone (*p* = 0.001) [34]. The alteration of the scoring system resulted in a different classification of the risk groups. This makes them difficult to compare, as shown in Table 1.

There has never been a prospective study comparing RIC allogeneic HSCT to consolidation with chemotherapy alone, but a retrospective comparison of RIC allo-HSCT with chemotherapy in patients aged 60–70 years with AML in first complete remission (CR) showed that allo-HSCT was associated with a significantly lower risk of relapse (32% vs. 81% at 3 years, *p* < 0.001), higher non-relapse mortality (NRM) (36% vs. 4% at three years, *p* < 0.001), and longer disease free survival (DFS) (32% vs. 15% at three years, *p* = 0.001), respectively [35].

An ongoing prospective randomized phase III study comparing conventional chemotherapy to allogeneic HSCT for older AML patients in first CR will hopefully bring clarity to the dilemma of decision-making whether or not to transplant elderly AML patients. Importantly, this data should help to prevent an undertreated population which is not offered curative intent treatment (https://clinicaltrials.gov/ct2/show/NCT00766779).

## 3. Bridging Strategies

Since allogeneic transplant is the only curative option for AML patients, including elderly patients, the major challenge is how to bridge them to transplant while on the one hand obtaining remission, but also maintaining good condition and avoiding severe complications. As with younger patients, a prerequisite for long-term survival of elderly AML patients undergoing allo-HSCT is achievement of CR before allo-HSCT [36]. However, treatment options and limitations for remission induction differ from those of young AML patients. Fatal toxicity of intensive induction therapy can bar the way to transplant. Moreover, patients with sAML or complex karyotypes tend to be refractory to standard induction therapy. Thus, a precise assessment whether the patient is considered to be a candidate for intensive chemotherapy or for new substances—such as azacitidine, decitabine, CPX-351 or venetoclax including biological risk stratification, as well as characteristics of the individual patient—is crucial for their outcome [21].

Bridging strategies can be divided into three broad categories: (a) diagnosis to CR (i.e., remission induction strategies); (b) CR1 to transplant (i.e., post-remission strategies); and (c) relapse/refractory to CR2 (i.e., salvage strategies) as well as strategies for allogeneic transplant in the absence of CR.

### 3.1. Remission Induction Strategies: Bridging from Diagnosis to CR

#### 3.1.1. Conventional Remission Induction

Elderly AML patients with intermediate or high-risk features and high WBC counts or high cellular turnover rates are generally considered candidates for conventional induction chemotherapy. Juliusson et al. retrospectively analyzed 2767 AML patients in the period from 1997 to 2005 covered by the Swedish Acute Leukemia Registry and clearly demonstrated that CR rates with intensive treatment are steadily decreasing due to increasing age and lower ECOG/WHO performance status [1]. Several studies such as the AML14 trial confirm CR rates for the elderly AML patient cohort of 40–60%, which have remained so for several decades, when being treated with the standard “3 + 7” regimens: i.e., three days of an anthracycline and seven days of cytarabine [21,37]. Obviously, the lower CR rates seen with conventional chemotherapy within the cohort of older compared to younger AML patients result from the higher frequency of poor cytogenetic and molecular risk factors, but also from patient-related factors [1,7,8,38]. However, the analysis of Juliusson et al. also provides data that standard intensive treatment–compared to palliation–improves long-term survival and early death rates. Interestingly, AML patients even up to 80 years seem to benefit from intensive induction chemotherapy, even with a poor initial performance status if it is considered disease-related [1,36]. Another small randomized phase III study by the European Organisation for Research and Treatment of Cancer (EORTC) was published in 1989, in which AML patients aged 65 years or above also had a better outcome in the intensive chemotherapy group than in the “wait-and-see” strategy group [39]. Nevertheless, prognosis of elderly AML patients with a median OS of less than one year still needs significant improvement. This fatal OS rate is also caused by an increased early death rate in patients aged 60 years or older compared to younger patients receiving intensive therapy [40,41,42,43]. Additionally, side effects of intensive induction therapy, especially severe infections during prolonged neutropenia and organ toxicities can disqualify the patient for other lines of therapy, in particular allo-HSCT. To more clearly identify patients for whom intensive chemotherapy is found to be suitable, the “AML-score” as a predictive algorithm was developed by Krug et al. This easily applicable method takes into account the probability of achieving CR by intensive treatment and the risk of early death for older patients with AML and has proven to be a very helpful tool [44].

#### 3.1.2. Anthracycline Dosing Considerations

There has been some debate in the past on the optimal dose of daunorubicin in induction. A randomized trial in elderly AML patients comparing 45 mg/m^2^ to 90 mg/m^2^ daunorubicin showed an increased rate of remission for the higher daunorubicin dose [45]. However, the more common regimen used in Europe is 60 mg/m^2^ daunorubicin, which has been compared to 90 mg/m^2^ in a large British trial of largely younger AML patients. In this trial, toxicity was higher in the 90 mg/m^2^ arm without an increase in CR rate. Thus, in our institution, we routinely employ 60 mg/m^2^ daunorubicin for remission induction regardless of age [46].

#### 3.1.3. Cytarabine Dosing Considerations

Similarly, different doss of cytarabine have been employed for induction therapy. Both 100 mg/m^2^ and 200 mg/m^2^ over seven days are commonly employed doses. In general, cytarabine doses above 1000 mg/m^2^ should not be included in induction regimens [21,47,48]. We prefer a dose of 100 mg/m^2^ d1–7 for patients >65 years.

#### 3.1.4. Targeted Therapies in Combination with Induction Chemotherapy

Elderly AML patients with a FLT3 mutation chemotherapy (*FLT3*-ITD or TKD) eligible for intensive should received midostaurin in addition to intensive induction [49]. To date, this is the only tyrosine kinase inhibitor that has shown a survival benefit in addition to 3 + 7 chemotherapy for FLT3-mutated AML. Addition of other targeted therapies such as IDH1/2 inhibitors to 3 + 7 is being evaluated in ongoing clinical trials and not part of standard care at this time.

#### 3.1.5. Novel Alternatives to Conventional Remission Induction Hypomethylating Agents

Highest allover CR rates are obtained with intensive induction chemotherapy, but regarding the heterogeneity of the diseases genetics (normal vs. complex karyotype), origin (de-novo vs. secondary), and dynamics (highly proliferative vs. smoldering), this is not valid for certain subgroups. Patients with a complex karyotype show CR rates of only about 30% in response to conventional induction therapy [22,50,51]. Accordingly, secondary AML response less favorably to standard induction than de-novo AML [22]. In contrast, these disease types show similar response rates under treatment with hypomethylating agents (HMAs) such as azacitidine and decitabine, regardless of cytogenetic risk category and even of TP53 status [51,52,53,54]. This most likely reflects the fact that sAML, often with complex karyotype or myelodysplasia-related changes, is biologically much more similar to MDS than to de-novo AML.

Thus, for these patients and those who are considered unlikely to reach CR with intensive chemotherapy while not presenting signs of highly proliferative disease hypomethylating agents (HMA) are reasonable alternatives to reach allogeneic HSCT (for an overview on selected novel bridging to transplant strategies see Table 2).

The efficacy of treatment with HMA in AML has been evaluated in several randomized trials. The DACO-16 trial randomizing 485 AML patients aged ≥65 years tested decitabine against BSC or LDAC. Decitabine significantly improved response rates compared to BSC/LDAC although OS was not significantly improved [55]. In a study by Blum et al., 47% of the patients achieved CR under treatment with decitabine. Notably, of those with a complex karyotype, 50% reached complete remission [56]. Correspondingly, Welch et al. described CR rates of 46% with higher response rates in the cytogenetic unfavorable risk group (67%) and with TP53 mutations (100%) [54]. In the setting of pretreatment before allo-HCT, a small trial by Lübbert et al. evaluated decitabine as induction therapy for older MDS and AML patients. 14/15 (93%) patients achieved complete remission, while 6/14 patients (42%) achieved longtime survival [57]. Various dosing schedules of decitabine have been evaluated for AML and the optimal schedule of decitabine is still a matter of debate.

The large randomized phase III trial AZA-AML-001 in AML patients older than 65 years with >30% blasts compared azacitidine with investigator’s choice of three conventional care regimens (LDAC, 3 + 7 or BSC only). Patients with adverse cytogenetics or myelodysplasia-related changes treated with azacitidine had an improved overall survival (6.4 vs. 3.2 months, *p* = 0.0185 and 12.7 vs. 6.3 months, *p* = 0.036, respectively) [58]. Notably, a nominal survival benefit was also seen for azacitidine in the absence of CR. In contrast, the evaluation of azacitidine as an addition to standard intensive chemotherapy 3 + 7 has not demonstrated improvement of OS but caused increased toxicity [59].

As a bridging therapy to allo-HSCT, most trials include high-risk MDS as well as sAML and show similar results for both entities. After first-line azacitidine application in high-risk MDS and AML patients, HSCT was feasible in the majority of patients with a pre-transplant CR rate of 24% and a median overall survival of 20.9 months after HSCT [60]. Interestingly, pre-transplant azacitidine application appears to decrease the risk of severe GvHD and to reduce the risk of relapse after HSCT compared to standard induction [61,62].

A novel second generation HMA guadecitabine (SGI-110) has also shown promising results in treatment-naïve AML patients ≥65 years [63]. In an open-label phase I/II study, the recommended regimen of guadecitabine was 60 mg/m^2^ over five days, resulting in a high composite CR rate of 54% (defined as CR, CR with incomplete platelet, or neutrophil recovery). These preliminary results are currently being further evaluated in a randomized phase III trial compared to standard of care.

#### 3.1.6. CPX-351

A novel cytotoxic chemotherapy approach that has yielded encouraging results is administration of CPX-351, the liposome-encapsulated formulation of cytarabine and daunorubicin at a fixed synergistic 5:1 molar ratio [68]. A randomized phase II trial tested CPX-351 (100 U/m^2^, which is equivalent to 100 mg/m^2^ cytarabine and 44 mg/m^2^ daunorubicin) against the conventional 3 + 7 regimen (100 mg/m^2^ cytarabine and 60 mg/m^2^ daunorubicin) as first-line treatment in 126 AML patients aged 60–75 years eligible for intensive chemotherapy [64]. Overall, CPX-351 produced higher response rates (66.7% vs. 51.2%, *p* = 0.07) with no significant differences for event-free survival (EFS) or OS. However, the subgroup analysis of patients with secondary AML demonstrated an improved response rate (57.6 vs. 31.6%, *p* = 0.06), with prolonged EFS (*p* = 0.08) and OS (*p* = 0.01). Importantly, more elderly AML patients reached allogeneic HSCT as a consequence in the CPX-351 arm. Additionally, Cortes et al. showed in a separate phase II trial of CPX-351 liposome injection vs. intensive salvage therapy in adults with first relapse a beneficial effect of the agent in the poor-risk stratum determined by the European Prognostic Index (EPI) [69]. Based on these results, a subsequent phase III trial was initiated and randomized 309 high-risk sAML patients aged 60 to 75 years to CPX-351 or 3 + 7. Treatment with CPX-351 resulted in longer OS (median, 9.6 vs. 6 months, *p* = 0.005; and two-year survival rates of 31% and 12%), lower 60-day mortality (13.7% vs. 21.2%) and improved response rates (CR/CRi 47.7% vs. 33.3%, *p* = 0.016) [65]. Thus, CPX-351 may improve treatment of fit older AML patients with high-risk features and provide an effective bridge to allogeneic HSCT. The FDA has approved CPX-351 for adult patients with newly diagnosed therapy-related AML or AML with myelodysplasia related changes.

#### 3.1.7. Venetoclax

The Bcl-2 inhibitor venetoclax has recently been granted breakthrough designation by the FDA based on very promising clinical results in combination with either hypomethylating agents (decitabine or azacitidine) or low-dose cytarabine (LDAC) in Phase I trials [66,67]. The CR rate in 57 newly diagnosed elderly AML patients was 61% for the combination of venetoclax and HMA, which is double that observed with HMA alone. Toxicity was in an acceptable range with a 30-day mortality below 10%. Remarkably, 9/57 (15.7%) patients enrolled in the trial of venetoclax + HMA subsequently underwent successful allo-HSCT while in remission, in part due to an improved clinical status, suggesting that this combination may be a feasible and useful bridging therapy to transplantation in elderly AML patients. Similarly, the CR/CRi rate in older AML patients treated with venetoclax + LDAC was 62% with a median CR/CRi duration of 14.9 months. However, these data are preliminary and the validation of a benefit of venetoclax combined with standard therapies in elderly patients with AML is currently ongoing (NCT02993523 and NCT03069352).

In summary, for patients in good condition with de-novo AML, high blast count, or a highly proliferative disease, we would recommend standard induction therapy with “3 + 7”. Patients with a complex karyotype or sAML might benefit more from pre-transplant treatment with HMA (+ venetoclax), reaching similar CR rates with less toxicity. CPX-351 has emerged as an interesting alternative for both groups. Continuing enrollment of elderly AML patients in clinical trials will be crucial to determine the optimal induction treatment for defined subgroups.

### 3.2. Post-Remission Strategies: Bridging from CR1 to Transplant

Even for intensively treated older AML patients the long-term survival (i.e., five years) is only approximately 10% and has not improved significantly in recent years compared to the progress which has been made in younger AML patients [8,37,70,71,72,73]. If achieved, maintaining remission in elderly patients after treatment with intensive or less-intensive induction chemotherapy remains a major issue, especially if a donor has not yet been defined.

Post-remission strategies comprising intensive chemotherapy and high- or intermediate-dose cytarabine (conventional intensive consolidation) followed by allogeneic HSCT as soon as possible should be aimed at whenever this is feasible. However, compared to younger AML patients this strategy addresses only a minority of elderly AML patients due to excessive toxicity and severe infections [21]. If further intensive consolidation after achieving CR is not possible due to toxicity or infectious complications, administration of azacitidine to maintain remission until alloHSCT is as a well-tolerated alternative [57,74].

The optimal post-remission therapy for older AML patients, as well as the optimal ‘bridge’ to transplant, remains unclear and hardly any adequate evidence addressing these issues exists. Despite the improvement of initial CR rates und extension of survival only a minority of these older AML patients starting with intensive or less-intensive induction chemotherapy reach allo-HSCT. Thus, we strongly recommend enrolling these patients in clinical trials. Innovative new approaches with more active and less toxic therapeutic agents are urgently needed for elderly AML patients to stabilize remissions more effectively and to successfully bridge to transplant.

#### Cytarabine Dosing Considerations

The optimal cytarabine dose for consolidation of CR in elderly AML patients has recently been called into question as new data has emerged. High-dose cytarabine with doses of 2000–3000 mg/m^2^ (3000 mg/m^2^ for younger patients) twice daily for three days has traditionally been employed as consolidation therapy, but is associated with increased hematologic, neurologic and gastrointestinal toxicities and may lie above the plateau of the maximal therapeutic effect [48]. Comparisons between high-dose cytarabine (HDAC) and intermediate dose cytarabine (IDAC, 1000–1500 mg/m^2^) or repeated cycles of conventional dose cytarabine, mostly in AML patients younger than 60–65 years, have failed to show an improved outcome (as measured by disease-free survival and overall survival) for HDAC [75,76]. Thus, HDAC is no longer routinely recommended for consolidation [21].

Two meta-analyses have examined published data on HDAC vs. IDAC for AML consolidation therapy [77,78]. Interpretation was difficult for both meta-analyses as trial design and cytarabine doses varied considerably between the included studies over a period of 25 years. However, although there was a risk reduction for HDAC in the favorable cytogenetics subgroup with an improved relapse-free survival, this was not observed for intermediate or high-risk cytogenetic subgroups. There was no benefit in terms of overall survival for HDAC in any of the cytogenetic risk groups compared to IDAC. Accordingly, the current ELN 2017 recommendations for AML patients >60/65 years include 2–3 cycles of consolidation with IDAC (500–1000 mg/m^2^ twice daily for 3 days or 500–1000 mg/m^2^ once daily for 5–6 days) for favorable risk cytogenetics. There is no clear established value of IDAC consolidation for intermediate and poor-risk subgroups in the older AML cohort [21]. However, in intermediate and high-risk AML patients deemed eligible for allo-HSCT, IDAC can successfully be employed as a bridge from CR to transplant while a donor search is ongoing.

### 3.3. Salvage Strategies: Bridging from Relapse or Refractory AML to Transplant

#### 3.3.1. Achievement of Second Complete Remission (CR2)

Since prognosis is dramatically reduced for AML patients undergoing allo-HSCT with active leukemia, an effective salvage chemotherapy is essential in refractory or relapsed disease. Facing the cumulative toxicity and the worsened prognosis, this is only feasible for patients in an outstandingly good condition and with a decided wish for a highly intensive treatment. In this case, conventional salvage therapy—such as high-dose cytarabine + mitoxantrone (HAM), intermediate-dose cytarabine + etoposide and mitoxantrone (MEC), or cytarabine + fludarabine and idarubicine (FLAG-IDA)—followed by reduced intensity conditioning and allogeneic HSCT is the procedure of choice in CR2 [79]. It is worth mentioning another approach to control refractory leukemia before allo-HSCT by sequential reduction of the leukemic burden with clofarabine, a second-generation purine analogue which is a highly effective drug in acute leukemia as experienced mainly in pediatrics, before conditioning is initiated [80,81,82].

Recently, concepts using novel substances to achieve a second remission are emerging. Gemtuzumab ozogamicin, a humanized antibody directed against CD33 and conjugated with the DNA toxin calicheamicin, has recently been re-approved by the FDA in combination with induction chemotherapy or as a single agent for treatment naïve AML patients and for relapsed refractory AML with high expression levels of CD33. A small sample size retrospective analysis by Cefalo et al. using fludarabine, cytarabine, and gemtuzumab ozogamicin (FLA-GO) as a bridge to transplant for relapsed/refractory adult AML patients suggests a favorable toxicity profile of this regimen [83]. However, a major caveat for patients receiving GO prior to alloHSCT is the risk of veno-occlusive disease (VOD), especially among patients who receive transplant within three months of GO administration. In addition, the highest benefit for addition of GO to chemotherapy was seen for AML with favorable or intermediate cytogenetics while the benefit for poor-risk cytogenetics is not clear [84]. Thus, the role for GO in bridging to alloHSCT currently remains undefined and further studies are required.

For relapsed/refractory AML patients with a targetable mutation such as *FLT3*-ITD or *IDH1/2*, administering small-molecule inhibitors may represent an alternative to conventional salvage chemotherapy. FLT3 tyrosine kinase inhibitors (TKI)—such as quizartinib, gilteritinib, and crenolanib—have activity as single agents in *FLT3*-mutated AML and several case studies showed that at least a number of elderly *FLT3*-ITD AML patients were able to be successfully bridged to alloHSCT by treatment with TKI monotherapy [85,86]. Accordingly, FLT3 inhibitors are currently being tested in combination with chemotherapy regimens such as MEC to improve CR rates in relapsed/refractory AML and have already shown promising results [87]. Similarly, IDH1/2 inhibitors have activity as single agents in relapsed and refractory AML and are currently also being tested in combination with intensive chemotherapy to improve CR rates [88].

For elderly AML patients achieving a remission, non-m≤yeloablative and reduced-intensity conditioning is the standard regimen. Various retrospective studies show encouraging data for the feasibility of RIC transplantation in patients >60 years [24,25,89,90]. In the first prospective multicenter phase II study by Devine et al. 114 AML patients in first CR with age 60 to 74 years underwent RIC-HSCT. Disease-free survival and OS at two years after transplantation were 42% and 48%, there was no difference between related and unrelated donors. NRM at two years was 15%, also independent of donor type. The incidence of relapse at two years was 44%, interestingly regardless of the cytogenetic risk profile (only 1 patient had favorable risk, 80 intermediate, 32 poor, and 1 unknown) [91].

In this study, fludarabine 30 mg/m^2^ for 5 days and busulfan 0.8 mg/kg for 8 doses on two days as one of the most common RIC protocols were administered, but conditioning regimens are used heterogeneously in different transplant centers. Besides fludarabine/busulfan, most common in the setting of CR are fludarabine/melphalan (≤140 mg/m^2^) and fludarabine/treosulfan (10 g/m²/day for three days), rarely fludarabine/melphalan/thiotepa. As recent data suggests, conditioning with fludarabine/treosulfan might have a benefit compared to fludarabine/busulfan. As presented by Beelen et al. in a prospective randomized multicenter trial designed to show at least non-inferiority of fludarabine/treosulfan conditioning in elderly MDS and AML patients, the EFS and estimated OS at two years after transplant was significantly higher in the treosulfan group (64.0% vs. 50.4% and 72.5% vs. 56.4%, respectively). Moreover, the treosulfan group showed a significantly lower TRM at two years of 11.3% compared to 28.2% [92].

#### 3.3.2. Strategies for Transplant in Absence of CR

For relapsed or refractory AML not reaching CR, the prognosis is generally extremely poor. Given the lack of comparative studies, the data on the combined FLAMSA-RIC (fludarabine/cytrabine/amsacrine) protocol appears encouraging for refractory AML patients with an OS of 40% at two years [93]. The conditioning part of FLAMSA-RIC can either be combined with 4 Gy total body irradiation (TBI) as in the original protocol, with busulfan, or, as recently published, with treosulfan. NRM was increased in the FLAMSA/treosulfan group (28% vs. 13% at four years) including patients with a significantly higher median age (60 vs. 46 years) and HCT-CI-score (2 vs. 0). However, the relapse rate in the group with blast persistence before protocol application was 35% in the treosulfan group versus 70% in the TBI group, suggesting a better leukemia control. OS and RFS were equal in both groups [94]. However, the benefit of allogeneic transplantation in a refractory situation is unclear in light of the low curative potential and must be weighed against the transplant-related mortality.

## 4. Outlook

Several feasibility studies clearly demonstrate the curative potential of allo-HSCT in elderly AML patients [38]. Unfortunately, the improvements achieved in the treatment of younger AML patients have not affected older patients yet. It has been shown that only approximately 32% of older AML patients starting with intensive chemotherapy reach transplant with continuous CR [36], but FDA approval of the four new drugs—midostaurin, enasidenib, CPX-351, and gemtuzumab ozogamicin—as well as the breakthrough designation for venetoclax for AML treatment in 2017 increases therapeutic options and brings new hope for the treatment of elderly AML patients as well [95]. Furthermore, the addition of the CXCR4-antagonist BL8040 to consolidation therapy as another new approach of targeted therapy is under investigation (NCT02502968).

Dual-affinity retargeting proteins (DARTS) or bispecific T-cell engager proteins (BiTES) represent novel immunologic therapies currently being evaluated for AML. Constructs which have shown promise in early clinical trials are the CD3 x CD33 construct and the CD3 x CD123 construct, which each target antigens highly expressed on AML cells [96,97]. Whether or not these antibody constructs will play a significant role as a bridging therapy to transplantation remains to be seen. Similarly, the role of chimeric antigen receptor T-cell (CAR-T) therapy, which has shown such promise in B-cell malignancies, is undefined for AML at the moment. A major obstacle for implementation of CAR-T cell therapy for AML will be identification of a suitable target on myeloid blast which does not severely affect normal hematopoiesis. Thus, if there is a future for CAR-T cell therapy as either bridging to allogeneic transplant or as an alternative procedure to transplant in AML remains to be determined.

In summary, new treatment algorithms are needed which consider specific risk models to identify the most beneficial treatment option for each individual of the highly heterogeneous cohort of elderly AML patients to improve long-term survival. Patients should be enrolled in clinical trials whenever possible.

## Figures and Tables

**Table 1:** Estimated two-year NRM in original and age-incorporating HCT-CI score. The original HCT-CI score is regardless of age, the estimated NRM by the HCT-CI/age score shown here is only based on the data of patients >60 years.

HCT-CI Score, Not Age Adapted [28]			HCT-CI/Age Score for Patient Group >60 Years [34]		
Score	Two-Year NRM	Risk	Score	Two-Year NRM	Risk
0	14%	Low risk	0	-	
1	22%	Intermediate risk	1–2	21%	Lower risk
2	19%		3–4	28%	Higher risk
3	41%	High risk	≥5	39%	(*p* = 0.02 and *p* < 0.0001)
≥4	40%				

HCT-CI vs. HCT-CI/age NRM.

**Table 2:** Selected novel bridging to transplant strategies for elderly AML patients.

Therapy	Reference	Type of Study	N	CR%	alloHCT%	PFS	OS	NRM%	Remarks
**Azacitidine**	[58]	Prospective, Phase III (Azacitidine vs. conventional care)	488 (241 Aza vs. 247 CC)	19.5% (+8.3% CRi)	-	6.7 months	10.4 months	16.2%	Selected patients not eligible for allo-HSCT
	[61]	Retrospective (Azacitidine pretreatment before allo-HCT in HR-MDS and sAML)	20	20%	100%	145 days	202 days	n.d.	Incidence of grade II to IV acute GVHD was significantly lower with Azacitidine pretreatment
	[60]	Prospective (Azacitidine followed by HSCT vs. no HSCT in MDS and AML)	97 (19 with AML)	24%	56%	n.d.	HSCT: 20.9 months; No HSCT: 9.4 months	n.d.	Azacitidine responders had a significantly longer survival than non-responders
	[62]	Retrospective (Azacitine vs. induction before allo-HCT in MDS and sAML)	68	n.d.	100%	n.d.	estimated 1-year OS 57%	n.d.	Pre-HSCT azacitidine led to a 66% lower hazard of relapse than conventional induction
**Decitabine**	[57]	Retrospective (Decitabine before allo-HSCT in MDS and AML)	15 (5 with AML)	33%	100%	n.d.	Longtime survival in 6 patients (40%), 2 with AML	33% (all transplant associated)	
	[55]	Prospective (Decitabine vs. conventional care)	485	27.7% (CR + CRi)	-	n.d.	7.7 months	24%	Selected patients not eligible for allo-HSCT
	[56]	Prospective, Phase II	53	47%	8%	55 weeks	46 weeks	15% (60 days)	CR rate of 50% in complex karyotypes
	[54]	Prospective	116	46%	n.d.	n.d.	n.d.	n.d.	CR rate of 67% in cytogenetic unfavorable risk group and 100% with TP53 mutations
**Guadecit abine**	[63]	Prospective, Phase II (5 or 10 day schedule of Guadecitabine)	107	50-59%	5%	n.d.	10.5 / 9.5 months	22%	Schedule of 60 mg/m^2^ on day 1–5 recommended
**CPX-351**	[64]	Prospective, Phase II (CPX-351 vs. 7 + 3)	126 (85 CPX-351, 41 7 + 3)	48,8% (+17.9% CRi)	16.5%	6.5 months	14.7 months	4.7% (60 days)	In sAML subgroup CPX-351 significantly improved OS (12.1 vs. 6.1 months)
**CPX-351** **Venetoclax and LDAC**	[65]	Prospective, Phase III (CPX-351 vs. 7 + 3)	309 (153 CPX-351, 156 7 + 3)	47.7% (CR + CRi)	n.d.	n.d.	9.56 months	13.7% (60 days)	
	[66]	Prospective, Phase Ib/II	71	62% (CR + CRi)	1%	n.d.	11.4 months	1%	
**Venetoclax and HMA**	[67]	Prospective, Phase Ib (Venetoclax + Decitabine or Azacitidine, dose escalation)	57	61% (CR + CRi)	18%	n.d.	12.3 months	7%	Equal response and safety profile in combination with azacitidine and decitabine

Selected novel bridging to transplant strategies for elderly AML patients.
